# The Single-Nucleotide Polymorphism of miR-27a *rs895819* and the Expression of miR-27a in *Helicobacter pylori*-Related Diseases and the Correlation with the Traditional Chinese Medicine Syndrome

**DOI:** 10.1155/2022/3086205

**Published:** 2022-03-15

**Authors:** Ling Zhang, Meng-Xin Huang, Dan-Yan Li, Yun-Zhan Zhang, Shao-Yang Lan, Qi Luo, Yun-Kai Dai, Yun-Bo Wu, Jin-Tong Ye, Wei-Jing Chen, Ru-Liu Li, Ling Hu

**Affiliations:** ^1^Institute of Gastroenterology, Science and Technology Innovation Center, Guangzhou University of Chinese Medicine, Guangzhou 510405, China; ^2^First Affiliated Hospital of Guangzhou University of Chinese Medicine, Guangzhou 510405, China

## Abstract

**Aims:**

The study aims to explore the effects of the single-nucleotide polymorphism of miR-27a and its expression in *Helicobacter pylori (H. pylori)*-related diseases and the relationship between gastric pathology and traditional Chinese medicine (TCM).

**Methods:**

Subjects were classified into six histopathological groups and five TCM syndrome groups. All specimens underwent *H. pylori* detection through rapid urease test and methylene blue staining. Histopathological characteristics were observed by hematoxylin-eosin. The expression of miR-27a and its genotype were, respectively, detected by Quantitative Real-Time PCR and direct sequencing.

**Results:**

*H. pylori* promoted the malignant evolution of gastric mucosa and were involved in the formation of TCM syndrome. In *H. pylori*-positive patients, the frequency of miR-27a CT genotype at the *rs895819* locus and its expression in the gastric cancer group were higher than those in other pathological groups. TCM syndrome had a close relationship with histopathological changes, and patients with spleen-qi deficiency syndrome had a higher risk of gastric cancer than other syndromes, regardless of *H. pylori* infection.

**Conclusion:**

The C allele at miR-27a *rs895819* locus may be an oncogene in gastric cancer. High levels of miR-27a could play an important role in gastric malignant evolution, especially cancerization. There is a certain connection between TCM syndrome and pathological changes of the gastric mucosa to some extent, where patients with SQD syndrome had a higher risk of GC.

## 1. Introduction

Gastric cancer (GC) is the fifth most common cancer and the third most common cause of cancer death worldwide, especially in Eastern Asian and Eastern European countries [1–4]. *Helicobacter pylori* (*H. pylori*), a type of Gram-negative bacteria, colonizes the epithelial cells of gastric mucosa and continuously releases toxins and induces inflammation. Chronic uncontrollable inflammation caused by *H. pylori* is the first step before carcinogenesis, which then develops into atrophy, intestinal metaplasia (IM), dysplasia, and eventually cancer [5–7]. The various lesions caused by *H. pylori*, covering the gastric mucosa from benign to malignant, are collectively referred to as H. *pylori*-related gastric disease (HPGD). The mechanism of HPGD is complicated and is related to the inflammation and immune response of gastric mucosa. In this process, the activation of related inflammatory pathways and the release of various inflammatory factors are the microscopic manifestations of gastric mucosal lesions.

MicroRNAs (miRNAs), a kind of small noncoding RNA, can modulate gene expression at the posttranscriptional level by either inhibiting messenger RNA (mRNA) translation or promoting mRNA degradation. miRNAs are highly related to many physiological and pathological processes, such as cancer, cardiovascular disease, digestive disorders, metabolic diseases, and neurodegeneration [8–11]. miR-27a, a member of the miRNA-27 family, plays a vital role in modulating polymorphisms, tumorigenesis, proliferation, apoptosis, invasion, migration, and angiogenesis [12]. Many studies have indicated that the overexpression of miR-27a can promote the proliferation and migration of tumor cells and enhance the drug resistance of GC [13–16]. Single-nucleotide polymorphism (SNP), a kind of DNA sequence polymorphism, refers to a variation of a single nucleotide in DNA, which is the most common type of human heritable variation, accounting for more than 90% of all known polymorphisms. SNP can reduce processing, lower levels, and disrupt the function of a mature miRNA by affecting RNA synthesis [17]. Arisawa et al. found that miR-27a polymorphism was associated with gastric mucosal atrophy [18]; Xu et al. revealed that miR-27a *rs895819* was involved in increased atrophic gastritis risk, improved gastric cancer prognosis, and negative interaction with *H. pylori* [19].

TCM syndrome is a comprehensive response of the body to internal and external pathogenic factors at a certain stage in diseases. Our previous research found that there was a close connection between TCM syndrome and HPGD. The TCM syndromes are a macroscopic manifestation of the pathological changes in gastric mucosa, which can reflect the pathological process of the gastric mucosa to a certain extent and can play a warning role in the evolution of gastric cancer [20–22]. Our study detected the SNPs of miR-27a *rs895819* and the miR-27a expression in different stages of HPGD to explore the microscopic differences of TCM syndromes and interpret the mechanism of pathological changes in gastric mucosa from multiple perspectives and thus can provide an early warning basis for the clinic.

## 2. Methods and Materials

### 2.1. Study Population

All subjects are from the Endoscopy Center and Gastrointestinal Surgery of the First Affiliated Hospital of Guangzhou University of Chinese Medicine from Oct 2016 to Oct 2017. The subjects were diagnosed with chronic gastritis (CG), peptic ulcer (PU), and GC. Four biopsy samples were obtained from the gastric antrum or mucosal lesions and used for rapid urease test, histopathology, miR-27a gene polymorphisms, and miR-27a detection. The research protocol was approved by the Ethics Committee of the First Affiliated Hospital of Guangzhou University of Chinese Medicine (ethical research permission code: No. [2015]009); all subjects signed informed consent.

### 2.2. *H. pylori* Detection and Criteria of Gastric Mucosal Histopathology

**Figure 1:**
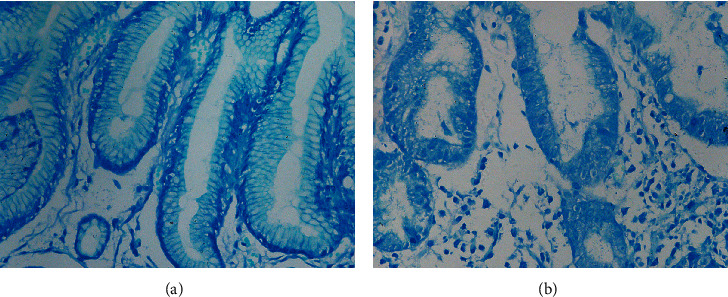
Representative images of *H. pylori* detection by methylene blue staining (magnification, ×400). (a) *H. pylori*-negative tissue and (b) *H. pylori*-positive tissue.

**Figure 2:**
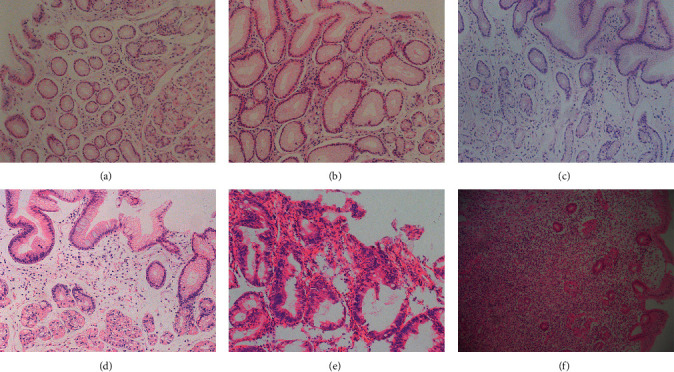
Representative images of different gastric histopathology (magnification, ×200). (a) Normal gastric mucosa; (b) gastric mucosa with moderate inflammation; (c) atrophic gastric mucosa with mild-to-moderate inflammation; (d) atrophic gastric mucosa with moderate-to-severe intestinal metaplasia; (e) severe dysplasia with mild inflammation, moderate atrophy, and intestinal metaplasia; and (f) gastric cancerous mucosa.

Criteria of *H. pylori* infection referenced to the Second Asia-Pacific Consensus Guidelines for *Helicobacter pylori* infection and Kyoto Global Consensus [23–25]. Rapid urease test (Guangzhou Beisiqi Reagent Co., Ltd., Guangdong, China) and methylene blue staining (Guangzhou Chemical Reagent Factory, Guangdong, China) were performed in all subjects, and either positive can be diagnosed as *H*. *pylori*-positive (Figure 1). Based on the distribution on the small curve on the surface epithelium, Hp infection can be divided into four grades [26]: (1) none: no Hp infection; (2) mild: occasionally or less the 1/3 length of the specimen; (3) moderate: continuously or accounting for 1/3–2/3 length of the specimen; (4) severe: diffusely or distributed over the entire length of the specimen. According to the updated Sydney System, consensus on chronic gastritis in China, and our research needs [22, 26, 27], specimens are classified into six groups (Figure 2): (1) normal group (NOR): normal histology or only mild inflammation; (2) inflammation group (INF): moderate-severe inflammation; (3) gastric atrophy group (GA): gastric gland atrophy without IM or dysplasia; (4) premalignant lesion group (PL): with IM or mild-moderate dysplasia; (5) severe dysplasia group (SD): severe dysplasia with gland atrophy and inflammation; (6) gastric cancer group (GC): different degrees of GC. In addition, we divided chronic inflammation, gastric atrophy, intestinal metaplasia, and dysplasia into four grades from no to severe, according to the updated Sydney System [26].

### 2.3. Criteria of TCM Syndromes

According to the syndrome differentiation in modern research of TCM and WHO International Standard Terminology on Traditional Medicine in the Western Pacific Region [28, 29], TCM syndromes of subjects were differentiated as follows: (1) nonsyndrome (NON): the subjects have no uncomfortable symptoms or abnormal signs; (2) spleen-stomach dampness-heat (SSDH) syndrome: a pathological condition ascribed to the accumulation of damp-heat that impairs the functions of the spleen and stomach, the same as dampness-heat in the middle energizer; (3) syndrome of liver-stomach disharmony (LSD): a syndrome marked by irritability, epigastric distension and pain, anorexia, belching, nausea, vomiting, and string-like pulse; (4) syndrome of spleen-qi deficiency (SQD): a pathological change characterized by qi deficiency with impaired transporting and transforming function of the spleen; (5) syndrome of internal blockade of static blood (IBSB): a pathological product of blood stagnation, including extravasated blood and blood circulating sluggishly or blood congested in a viscus, all of which may turn into a pathogenic factor, the same as blood stasis or stagnant blood. The TCM syndromes were diagnosed by two physicians and were confirmed in the condition of two primary symptoms or one primary symptom with two secondary symptoms.

### 2.4. Genotyping of miR-27a rs895819

**Figure 3:**
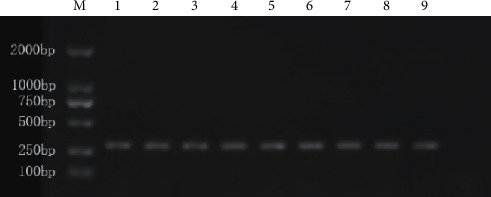
PCR-amplified fragments. Lane M is the marker; lanes 1 to 9 are the DNA regions (300 bp) containing the miR-27a (*rs895819*) from 9 different individuals.

HiPure Tissue DNA Kits (Magen, D3121-02) were used to extract DNA from gastric mucosal tissue. PCR amplification was performed by PCR DSMIX (Dongsheng Biotech, Guangdong, China; 077). The primer sequences were as follows: forward primer: 5′-TGTGTTTCAGCTCAGTAGGCAC-3′; reverse primer: 5′-CTGTCACAAATCACATTGCC-3′. The PCR conditions were 94°C for 4 min; then 40 cycles of 94°C for 30 s, 60°C for 30 s, and 72°C for 30 s; finally, 72°C for 10 min and 10°C 30 s. The PCR products were separated by 1% agarose gel electrophoresis (Figure 3). Then, the direct sequencing of PCR products was performed with the dideoxy-chain method.

### 2.5. miRNA-27a Expression Detection

HiPure FFPE miRNA Kits (Magen, R4313-02) were used to extract total RNA from gastric mucosal specimens, and then cDNA was reverse-transcribed and synthesized by ReverTra Ace qPCR RT Kits (ToYoBo, 511000). The cDNA was amplified by quantitative fluorescence PCR, and Typrobe, ROX dye, and KAPA Probe Fast qPCR Master Mix were used (KAPA Biosystems, KK4702). The conditions were 95°C for 2 min; 40 cycles of 95°C for 5 s, 55°C for 5 s, and 70°C for 30 s. The primer sequence is consistent with the above miRNA-27a genotyping primer sequence. U6 is the internal reference gene, and the expression difference of miR-27a was analyzed by the 2^−ΔΔCp^.

### 2.6. Statistical Analysis

SPSS 25.0 and Stata 15.0 software were used for statistical analysis. Categorical variables including gender, *H. pylori* infection rate, distribution of TCM syndrome, and genotype were summarized by proportions and were analyzed by the Chi-square test or Fisher's exact probability test. Levels of miR-27a were assessed by the Kruskal–Wallis H test due to unconformity with normal distribution. The miaR-27a *rs895819* genotype distribution was examined by the Hardy–Weinberg equilibrium. Multivariate logistic regression analysis was conducted to explore compound factors in the formation of gastric pathology and TCM syndrome. *P* value less than 0.05 indicated that the difference was statistically significant.

## 3. Results

### 3.1. Characteristics of Subjects

In total, 213 participants (129 males and 84 females) were included in the study. The distribution of age, gender, Hp infection, and miR-27a genotype of gastric pathological group and TCM syndrome group are displayed in Tables 1 and 2. Through the Fisher exact probability test, a significant difference in age and *H. pylori* infection among histopathological groups and TCM syndrome group was found (*P* < 0.05). Specifically, the proportion of patients over 65 in the GC group was higher than that of the INF and PL group; the *H. pylori* infection rate of the PL and GC group was greater than that of the NOR and INF groups. In the TCM syndrome group, both the SSDH and LSD groups had higher *H. pylori* infection rate than that in the NON and SQD groups, while it was higher in the SQD group than the NON group. All *P* values were less than 0.05.  Table 3 shows the distribution of age, gender, Hp infection, gastric pathology, and TCM syndrome in different genotypes. No statistical difference was found.

### 3.2. The Severity of *H. pylori* Infection

**Figure 4:**
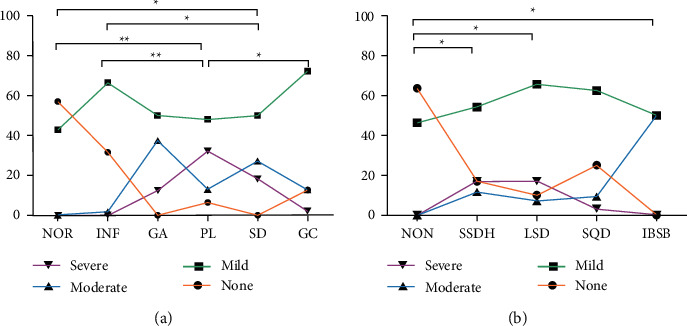
The situation of *H. pylori* infection in histopathology group and TCM syndrome group. (a) Histopathology groups; (b) TCM syndrome groups. ^∗^*P* < 0.05, ^∗∗^*P* ≤ 0.001.

As indicated in Figure 4(a), in the histopathological groups, the *H. pylori* infection degree in the PL and SD groups was higher than that in the NOR (*P* ≤ 0.001, *P* = 0.002) and INF groups (*P* ≤ 0.001, *P* *=* 0.007); the H. *pylori* infection in the PL group was more severe than that in the GC group (*P* = 0.008). Regarding the TCM syndrome groups (Figure 4(b)), SSDH, LSD, and IBSB groups had a higher H. *pylori* infection degree than the NON group (*P* = 0.003, 0.002, 0.016).

### 3.3. Relationship between Gastric Histopathology/TCM Syndrome

**Figure 5:**
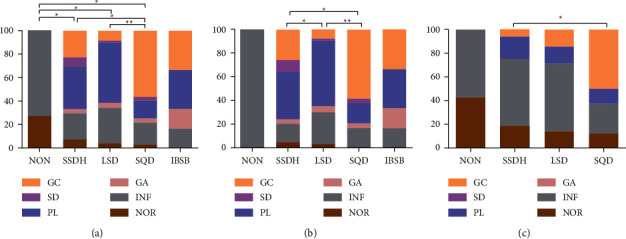
Relationship between gastric histopathology and TCM syndrome. (a) All subjects; (b) *H. pylori*-positive subjects; (c) *H. pylori*-negative subjects. ∗*P* < 0.05, ∗∗*P* ≤ 0.001.

Figure 5 illustrates the correlation between gastric histopathology and TCM syndrome. The distribution of gastric histopathology in NON, SSDH, LSD, and SQD syndrome groups was significantly different (*P* < 0.05). On the whole (Figure 5(a)), the ratio of the NOR group in the NON syndrome group was higher than that of the LSD syndrome group (27.3% vs. 4.3%), while the proportion of the INF group was far beyond the SSDH and SQD syndrome group (72.7% vs. 22.3%; 72.7% vs. 18.8%). Besides, PL was more common in LSD syndrome than in SQD syndrome (51.4% vs. 15.6%), and GC was more likely to occur in SQD syndrome than in SSDH and SD syndrome (56.3% vs. 22.3%; 56.3% vs. 15.6%). To further analyze the effect of *H. pylori* infection, we divided all subjects into *H. pylori*-positive and *H. pylori*-negative groups. In *H. pylori*-positive patients (Figure 5(b)), the proportion of GC in the SQD syndrome was much higher than that in the SSDH and LSD syndrome (58.3% vs. 25.6%; 58.3% vs. 8.6%), and GC in SSDH syndrome was more common than LSD syndrome (25.6% vs. 7.9%). Besides, the ratio of PL in SQD syndrome was lower than that of the LSD syndrome (16.7% vs. 55.6%). In H. *pylori-*negative subjects (Figure 5(c)), we also found the proportion of GC in SQD syndrome was remarkably higher than that in the SSDH syndrome (50.0% vs. 6.3%). All *P* values were less than 0.05.

### 3.4. The miR-27a rs895819 Locus SNPs in Gastric Histopathology/TCM Syndrome

**Figure 6:**
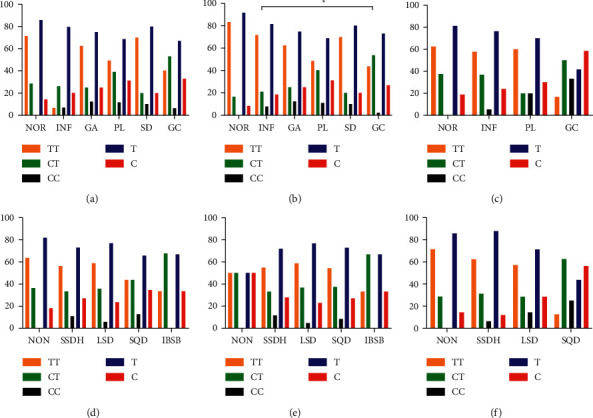
Distribution of genotype and allele frequency in histopathology and TCM syndrome groups. Histopathology groups in (a) all subjects, (b) *H. pylori*-positive subjects, and (c) *H. pylori-*negative subjects; in TCM syndromes groups in (d) all subjects, (e) *H. pylori-*positive subjects, and (f) *H. pylori*-negative subjects. ∗*P* < 0.05.

The frequency of TT, CT, and CC genotypes of miR-27a *rs895819* was 54.9% (117/213), 36.6% (78/213), and 8.5% (18/213), and the alleles did not deviate from the Hardy–Weinberg genetic equilibrium law.  Figure 6 reveals the distribution of TT, CT, and CC genotypes and C/T allele frequency in different pathological groups and TCM syndrome groups. No statistical difference was observed in the histopathological group, neither in the whole subjects nor the *H. pylori*-negative subjects (Figures 6(a) and 6(c)). However, in *H. pylori*-positive patients (Figure 6(b)), the distribution of genotype in INF group and GC group was significantly different, with the higher frequency of CT genotype in GC group compared to INF group (53.7% vs. 21.1%). The frequency of genotypes in TCM syndrome groups showed no statistical difference, regardless of *H. pylori* infection (Figures 6(e)–6(f)). However, among *H. pylori-*negative patients, the frequency of CT genotype in the SQD group tended to increase compared with other syndrome groups.

### 3.5. The Effect of miR-27a rs895819 Genotype on Its Expression

To explore the effect of *rs895819* genotype on miR-27a expression, we analyzed the levels of miR-27a in different genotypes in both the histopathological group and the TCM syndrome group (Table 4). Altogether, the expression of miR-27a in different genotypes showed no significant difference (*P* > 0.05). However, patients with CT genotype tended to express more miR-27a than those with TT/CC genotype. There was no statistical difference in miR-27a expression in different genotypes between each of the pathological groups and TCM syndrome groups as well (*P* > 0.05).

### 3.6. miR-27a Expression in Histopathological Group/TCM Syndrome Group

**Figure 7:**
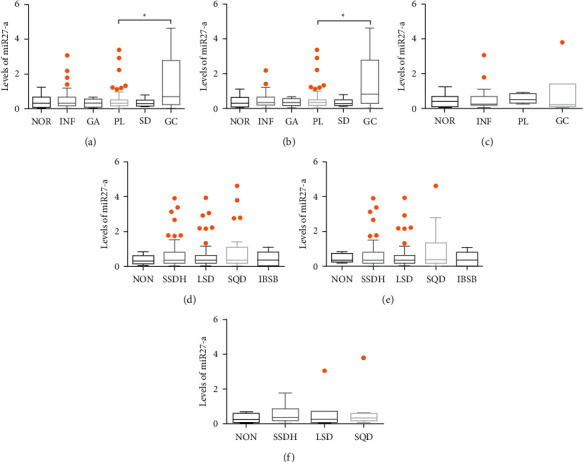
Effect of miR-27a expression in histopathology and TCM syndrome groups. miR-27a expression in histopathology groups in (a) all subjects, (b) *H. pylori-*positive subjects, and (c) *H. pylori*-negative subjects; miR-27a expression in TCM syndrome group in (d) all subjects, € *H. pylori*-positive subjects, and (f) *H. pylori*-negative subjects. ∗*P* < 0.05.

The levels of miR-27a in each histopathological group and TCM syndrome group are displayed in Figure 7. In all subjects (Figure 7(a)), miR-27a expression in the GC group was dramatically higher than that in the PL group (*P* = 0.043). For *H. pylori*-positive patients (Figure 7(b)), the GC group also had higher levels of miR-27a than the PL group (*P* = 0.004), and there was no significant difference in miR-27a expression for *H. pylori*-negative patients (Figure 7(c)). These results suggested that the occurrence of GC was associated with the high expression of miR-27a. Whether infected with *H. pylori* or not, the levels of miR-27a in the TCM syndrome group showed no statistical difference (Figures 7(d)–7(f)). Nevertheless, miR-27a expression in the SQD group tended to increase compared to other groups in *H. pylori*-positive patients.

### 3.7. The Correlation of miR-27a Level and Its Genotype with *H. pylori* Infection and Gastric Histopathology

**Figure 8:**
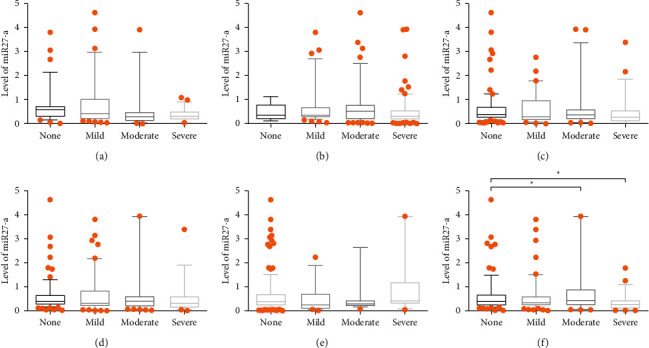
Correlation of miR-27a expression with the severity of *H. pylori* infection and the grade of histopathology. miR-27a expression in (a) grading of *H. pylori* infection; (b) grading of chronic inflammation; (c) grading of polymorphonuclear neutrophil activity; (d) grading of glandular atrophy; (e) grading of intestinal metaplasia; (f) grading of dysplasia. ^∗^*P* < 0.05.

**Figure 9:**
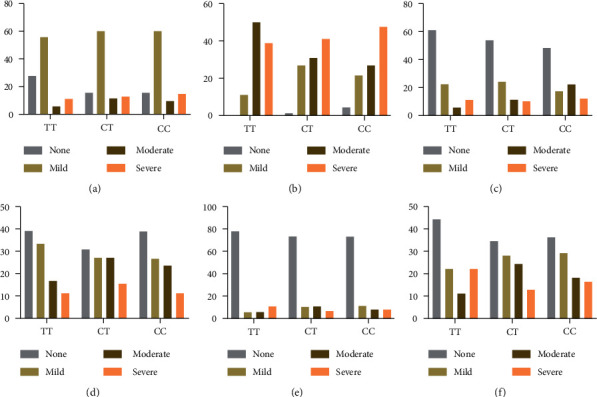
Correlation of miR-27a genotype distributions with the severity of *H. pylori* infection and the grade of histopathology. miR-27a genotype distributions in (a) grading of *H. pylori* infection; (b) grading of chronic inflammation; (c) grading of polymorphonuclear neutrophil activity; (d) grading of glandular atrophy; (e) grading of intestinal metaplasia; (f) grading of dysplasia.

Figure 8 reveals the relationship between miR-27a levels and the severity of gastric histopathology and *H. pylori* infection. The levels of miR-27a have no relationship with the severity of *H. pylori* infection and the grade of chronic gastric inflammation, gastric atrophy, and intestinal metaplasia. However, patients without gastric dysplasia expressed higher levels of miR-27a compared to those with moderate and severe dysplasia (*P* < 0.05). In addition, Spearman's analysis showed that the degree of *H. pylori* infection had a negative correlation with the levels of miR-27a (Table 5).  Figure 9 presents the relation between the miR-27a genotype and the severity of *H. pylori* infection and the grade of gastric histopathology. The results showed no statistical difference and Spearman's analysis (Table 5) showed no correlation between them (*P* > 0.05).

### 3.8. Multivariate Logistic Regression Analysis

To further evaluate the effect of compounding factors such as age, gender, *H. pylori*, and genotype, we performed a multivariate logistic regression analysis on gastric histopathology and TCM syndrome.  Table 6 describes the outcome of the multinomial logistic regression between covariables and gastric histopathology using the NOR group as the reference. Compared with the NOR group, the female gender decreased the risk of GC (OR = 0.23), and *H. pylori* dramatically increased the risk of PL (OR = 26.85) and GC (OR = 10.16). Besides, the CT genotype was correlated with a high risk of GC (OR = 5.03).  Table 7 shows the results of multinomial logistic regression between covariables and TCM syndrome using the NON group as the reference. Compared with the NON group, *H. pylori* infection significantly elevated the risk of SSDH (OR = 9.69), LSD (OR = 20.98), and SQD group (OR = 5.33). In addition, ages above 65 reduced the risk of LSD (OR = 0.09), while ages between 45 and 55 decreased the risk of SQD (OR = 0.1). After adjusting for age, gender, and *H. pylori*, no significant relation was found between TCM syndrome and miR-27a *rs895819* SNPs.

## 4. Discussion

This study revealed that age, gender, levels of miR-27a, and its SNPs were closely related to HPGD, and *H. pylori* played a crucial role in the development of HPGD. *H. pylori* colonize the surface of the gastric mucosa by secretion of urease and then release virulence factors such as OpiA, CagA, and VacA, which cause damage to gastric mucosa persistently [30]. Our study showed that the infection rate of *H. pylori* and the severity of infection gradually elevated with the malignant evolution of gastric mucosal. Furthermore, the multivariate logistic regression analysis showed that H. *pylori* infection could dramatically increase the risk of PL and GC (OR = 26.85, 10.16), which indicated that *H. pylori* played a major role in HPGD. At the same time, plenty of studies showed that the eradication of *H. pylori* might decrease the risk of gastric atrophy, intestinal metaplasia, dysplasia, and carcinoma [31–33].

Around 41.35–72.3% of Chinese adults were infected with *H. pylori* [34], whereas only a part of them developed into the disease, which may be related to multiple factors such as the virulence of *H. pylori*, the host immune response, lifestyle, and the environmental factors [35, 36]. Our study found that patients in the GC group were older than those in the INF group, and as age increased, the probability of malignant transformation of gastric mucosa also increased, which may be due to the lower ability of immune response and self-repair and the higher infection rate and severity of *H. pylori*. de Vries et al. discovered that the risk of GA in patients over 40 was twice that of those under 40 [36]. A study also showed that older age was one of the risk factors in GC [4]. Moreover, gender can also influence the occurrence of the disease. According to the global survey, the proportion of males in GC was twice as much as that of females [2]. In this study, we found that the female gender had a lower risk of GC than the male gender (OR = 0.23), which was probably related to the unhealthy lifestyle in males, such as drinking and smoking, and these were considered risk factors of GC [4].

It has been found that miR-27a was closely correlated to GC. Zhang et al. demonstrated that miR-27 could induce epithelial-to-mesenchymal transition by activating the Wnt pathway and thus promoting gastric cancer cell metastasis [15]. Ding et al. discovered that miR-27a promoted the proliferation and metastasis of gastric tumor cells by suppressing PHLPP2 and activating the AKT/GSK3*β* pathway [13]. In this study, we detected the expression of miR-27a in each pathological group. No significant difference in miR-27a expression was found from NOR to SD group. However, when gastric mucosa became cancerous, the expression of miR-27a was dramatically increased regardless of *H. pylori* infection. Therefore, we speculated that the expression of miR-27a was tightly connected with the development of GC. Interestingly, our study also found that miR-27a expression has a negative correlation with the severity of *H. pylori* infection.

Furthermore, our study also analyzed the effect of miR-27a *rs895819* polymorphism on HPGD. Although the distribution of TT/CT/CC genotype and the frequency of alleles showed no statistical difference among the histopathological groups in *H. pylori*-negative patients, the proportion of CC genotype and C allele tended to increase with the malignant evolution of gastric mucosa. In *H. pylori*-positive patients, the ratio of CT genotype in the GC group was significantly higher than that in the INF group. Also, the multivariate logistics regression analysis showed that the CT genotype dramatically increased the risk of GC (OR = 5.30). Therefore, we speculated that the C allele was an oncogene in GC, and patients with CT genotype have a high risk of malignant changes of the gastric mucosa.

To study the relation between miR-27a genotype and its expression, we also detected the expression of miR-27a in different genotypes. The level of miR-27a in the CT genotype tended to increase compared with TT/CC group, which is consistent with Xu et al.' study [19]. Consequently, we speculate that the SNPs of *rs895819* may affect miR-27a expression to some extent.

The syndrome is the core of TCM, and its formation is the result of the combination of multiple factors such as age, diet, living style, external evil (i.e., *H. pylori*), and body's health condition. We explored the relationship between TCM syndrome and *H. pylori* infection, gastric histopathology, and miR-27a expression. We discovered that the TCM syndromes could reflect pathological changes to a certain extent. Among *H. pylori*-positive patients, the gastric mucosal of the NON group was normal or showed only mild inflammation. Patients with SSDH or LSD syndrome were likely to develop gastric atrophy or intestinal metaplasia, while GC was most common in the SQD group. Without *H. pylori* infection, most of the patients in the NON, SSDH, and LSD groups showed only inflammation, but the proportion of GC still took advantage in the SQD group. Meanwhile, the *H. pylori* infection rate and infection degree of the SSDH, LSD, SQD, and IBSB groups were much higher than those of the NON group. Therefore, we presume that TCM syndrome is an external manifestation of gastric mucosal lesions and *H. pylori* infection. The combination of TCM syndrome and *H. pylori* could help infer the histopathological changes of gastric mucosa clinically. At the molecular level, we found no statistical differences in the distribution of miR-27a genotypes and miR-27a expression in the TCM syndrome group. However, the proportion of CT genotype in the SQD group tended to increase among *H. pylori*-negative patients. The expression of miR-27a in the SQD group seemed to be higher than other syndrome groups among *H. pylori*-positive patients. It is reasonable to speculate that miR-27a and its SNPs play a role in the formation of SQD.

In this study, we found that whether infected with *H. pylori* or not, patients with SQD had a higher risk of GC than that of other syndromes, which suggested that patients with GC were likely to present as SQD. Many scholars have studied the gastric pathological changes in SQD. Liu et al. found that the gastric mucosal morphology and blood flow were changed, and the expression of multiple immune factors was reduced in SQD model rats [37]. Under the transmission electron microscope, Hu et al. discovered significant changes of ultrastructure in gastric mucosal epithelium cells in SQD patients, with the overexpression of MUC5A in crypt gland cells [21]. *H. pylori* can release various virulence factors to activate cell signaling pathways such as PI3-kinase/Akt, JAK/STAT and Ras, Raf, and ERK signaling pathways [38] and promote the expression of multiple carcinogenic factors such as miR-27a, eventually leading to cell cancerization. Cancer cells proliferate and spread and continue to damage the gastric mucosa and surrounding tissue. The early manifestations of GC are simply a series of dyspeptic symptoms such as abdominal distention and loss of appetite, which are consistent with the clinical symptoms of SQD. Clinical studies have shown that Jianpi-Yiqi medicine combined with chemotherapy can alleviate clinical symptoms, improve the quality of life, reduce adverse reactions, and enhance the immune function in GC patients [39]. Therefore, patients with *H. pylori* infection, clinically manifested as SQD syndrome, who have unhealthy lifestyles such as drinking and smoking, should have a gastroscope examination and early cancer screening.

Our study has several limitations. First, there were only a few subjects in the GA and SD groups in our study, affecting the conclusion. In addition, the patients included were all Chinese and from the same hospital. Hence, the external validity still needs to be studied on a large scale and in different races.

## 5. Conclusion

This study indicates that male gender, aging, and Hp infection are risk factors in the formation of gastric precancerous lesions and GC. The C allele at miR-27a *rs895819* locus may be a susceptibility gene for GC. The high level of miR-27a expression may promote the occurrence of GC and SQD syndrome to a certain extent. There is a certain connection between TCM syndrome and pathological changes of the gastric mucosa, and patients with SQD syndrome are more likely to have gastric cancerization. The combination of *H. pylori* detection, TCM syndrome, the CT genotype at miR-27a *rs895819* and its high expression may have a warning effect on gastric mucosal lesions [35].

## Figures and Tables

**Table 1:** Distribution of age, gender, *H. pylori*, and miR-27a genotypes in gastric histopathological groups.

	All (*N* = 213) wt. %	NOR (*n* = 14) wt. %	INF (*n* = 57) wt. %	GA (*n* = 8) wt. %	PL (*n* = 77) wt.%	SD (*n* = 10) wt.%	GC (*n* = 47) wt.%	*P*
Age								
<45 years	36.2	42.9	40.4	37.5	39.0	10.0	29.8	**0.041**
45∼years	28.2	21.4	36.8	25.0	32.5	30.0	12.8	
55∼years	21.6	21.4	12.3	25.0	20.8	40.0	29.8	
65∼years	14.1	14.3	10.5	12.5	7.8	20.0	27.7	
Gender								
Male	60.6	42.9	53.7	55.6	61.5	63.6	72.3	0.303
Female	39.4	57.1	46.3	44.4	38.5	36.4	27.7	
*H*. *pylori*								
Negative	17.8	57.1	33.3	0	6.5	0	12.8	**0.001**
Positive	82.2	42.9	66.7	100	93.5	100	87.2	
miR-27a genotype								
TT	54.9	57.1	61.1	55.6	50.0	72.7	51.1	0.149
CT	36.6	28.6	26.3	25.0	39.0	20.0	53.2	
CC	8.5	0.0	7.0	12.5	11.7	10.0	6.4	

NOR = normal group; INF = inflammation group; GA = gastric atrophy group; PL = premalignant lesion group; SD = severe dysplasia group; GC = gastric cancer group. The bold values mean that the distribution of age and miR-27a genotype in different pathological groups are statistically different.

**Table 2:** Distribution of age, gender, *H. pylori*, and miR-27a genotypes in TCM syndrome groups.

	All (*N* = 213) wt. %	NON (*n* = 11) wt. %	SSDH (*n* = 94) wt. %	LSD (*n* = 70) wt. %	SQD (*n* = 32) wt.%	IBSB (*n* = 6) wt.%	*P*
Age							
<45 years	36.2	18.2	36.2	41.4	31.3	33.3	**0.018**
45∼years	28.2	45.5	27.7	35.7	9.4	16.7	
55∼years	21.6	9.1	23.4	14.3	31.3	50.0	
65∼years	14.1	27.3	12.8	8.6	28.1	0.0	
Gender							
Male	60.6	72.7	67.7	53.5	56.3	33.3	0.159
Female	39.4	27.3	32.3	46.5	43.8	66.7	
*H. pylori*							
Negative	17.8	63.6	17.0	10.0	25.0	0.0	**0.001**
Positive	82.2	36.4	83.0	90.0	75.0	100.0	
miR-27a genotype							
TT	54.9	45.5	54.9	60.6	46.9	50.0	0.616
CT	36.6	36.4	33.0	35.7	43.8	66.7	
CC	8.5	0.0	10.6	5.7	12.5	0.0	

NON = nonsyndrome; SSDH = syndrome of spleen-stomach dampness-heat; LSD = syndrome of liver-stomach disharmony; SQD = syndrome of spleen-qi deficiency; IBSB = syndrome of internal blockade of static blood. The bold values mean that the distribution of age and *H*. pylori infection rate in different TCM syndrome groups is statistically different.

**Table 3:** The basic characteristic of subjects based on miR-27a genotypes.

	TT (*N* = 117) wt.%	CT/CC (*N* = 96) wt.%	*P* value
Age			
<45 years	40.2	31.3	0.592
45∼years	25.6	31.3	
55∼years	20.5	22.9	
65∼years	13.7	14.6	
Gender			
Male	64.1	56.3	0.262
Female	35.9	43.8	
*H. pylori*			
Negative	15.4	17.7	0.712
Positive	84.6	82.3	
Histopathology			
NOR	6.8	6.3	0.659
INF	28.2	21.9	
GA	4.3	4.2	
PL	33.3	40.6	
SD	6.8	3.1	
GC	20.5	24.0	
TCM syndrome			
NON	4.3	6.3	0.695
DHSS	43.6	43.8	
GWD	36.8	29.2	
SQD	12.8	17.7	
IBSB	2.6	3.1	

**Table 4:** The effect of miR-27a *rs895819* polymorphism on its expression.

	TT		CT		CC		*P*
	*N*	Mean ± SD	*N*	Mean ± SD	*N*	Mean ± SD	
Total	117	0.62 ± 0.96	78	1.19 ± 2.28	18	0.43 ± 0.30	0.980

Histological types							
NOR	10	0.46 ± 0.42	4	0.38 ± 0.36	0	—	0.777
INF	38	0.42 ± 0.31	15	0.79 ± 0.92	4	0.27 ± 0.10	0.746
GA	5	0.27 ± 0.21	2	0.33 ± 0.35	1	0.67	0.303
PL	38	0.45 ± 0.41	30	0.57 ± 0.77	9	0.42 ± 0.35	0.948
SD	7	0.34 ± 0.23	2	0.29 ± 0.16	1	0.59	0.452
GC	19	1.67 ± 1.99	25	2.44 ± 3.61	3	0.53 ± 0.38	0.671
*P*	0.793			0.363	0.408		

TCM syndromes							
NON	7	0.31 ± 0.20	4	0.50 ± 0.35	0	—	0.344
DHSS	53	0.61 ± 0.71	31	1.33 ± 2.91	10	0.46 ± 0.36	0902
LSD	41	0.58 ± 0.71	25	0.96 ± 1.77	4	0.30 ± 0.19	0.584
SQD	14	0.96 ± 2.11	14	1.71 ± 2.14	4	0.49 ± 0.20	0.809
IBSB	2	0.55 ± 0.76	4	0.37 ± 0.28	0	—	1.000
*P*	0.476		0.083		0.491		

**Table 5:** Correlation analysis between levels of miR-27a/miR-27a genotype and the severity of *H. pylori* infection and the grade of histopathology.

	Levels of miR-27a		miR-27a genotype	
	Spearman's	*P*	Spearman's	*P*
Hp infection	**−0.175**	**0.012**	0.035	0.614
Gastric inflammation	−0.112	0.102	0.019	0.570
Inflammatory activity	−0.122	0.076	0.094	0.171
Gastric atrophy	−0.086	0.209	−0.068	0.326
Gastric intestinal metaplasia	−0.016	0.811	0.014	0.845
Gastric dysplasia	−0.117	0.089	0.002	0.972

The bold values mean that the levels of miR-27a have a negative correlation with Hp infection, and the correlation index is ‐0.175, which is statistically significant.

**Table 6:** Multivariate logistic regression analysis in pathological groups.

	INF vs. NOR		GA vs. NOR		PL vs. NOR		SD vs. NOR		GC vs. NOR	
	OR (95% CI)	*P*	OR (95% CI)	*P*	OR (95% CI)	*P*	OR (95% CI)	*P*	OR (95% CI)	*P*
Gender										
Male	Ref	—	Ref	—	Ref	—	Ref	—	Ref	—
Female	0.53 (0.15–1.89)	0.332	0.63 (0.99–4.01)	0.625	0.37 (0.10–1.36)	0.134	0.40 (0.67–2.40)	0.319	**0.23 (0.05–0.89)**	**0.033**

*H. pylori*										
Negative	Ref	—	Ref	—	Ref	-	Ref	—	Ref	—
Positive	3.32 (0.95–11.65)	0.060	—	0.995	**26.85 (6.09–118.35)**	**0.001**	—	0.994	**10.16 (2.38–43.43)**	**0.002**

Age										
≤45	Ref	—	Ref	—	Ref	—	Ref	—	Ref	—
45∼54	1.91 (0.40–9.02)	0.414	1.41 (0.13–15.09)	0.775	1.77 (0.35–8.85)	0.486	7.05 (0.46–109.74)	0.161	0.91 (0.15–5.42)	0.915
55∼64	0.49 (0.90–2.71)	0.417	0.85 (0.76–9.45)	0.893	0.71 (0.13–3.84)	0.691	5.54 (0.37–82.90)	0.215	1.47 (0.26–8.34)	0.662
≥65	0.62 (0.93–4.16)	0.624	0.78 (0.43–14.11)	0.869	0.46 (0.64–3.32)	0.442	4.93 (0.25–98.51)	0.296	2.23 (0.32–15.15)	0.410

miR-27a (*rs895819*) genotype										
TT	Ref	—	Ref	—	Ref	—	Ref	—	Ref	-
CT	1.26 (0.31–5.18)	0.745	1.57 (0.18–13.33)	0.678	3.00 (0.72–12.56)	0.132	1.03 (0.13–8.31)	0.981	**5.30 (1.21–23.15)**	**0.027**
CC	—	—	—	—	—	—	—	—	—	—
T	Ref	—	Ref	—	Ref	—	Ref	—	Ref	—
C	1.1 (0.33–3.61)	0.875	1.17 (0.19–7.21)	0.868	0.69 (0.22–2.13)	0.518	1.50 (0.25–9.11)	0.660	0.46 (0.15–1.46)	0.187

All the bold values represent that the outcome was statistically significant.

**Table 7:** Multivariate logistic regression analysis in TCM syndrome groups.

	SSDH vs. NON		LSD vs. NON		SQD vs. NON		IBSB vs. NON	
	OR (95% CI)	*P*	OR (95% CI)	*P*	OR (95% CI)	*P*	OR (95% CI)	*P*
Gender								
Male	Ref	—	Ref	—	Ref	—	Ref	—
Female	1.42 (0.31–6.54)	0.649	2.78 (0.59–13.14)	0.198	2.30 (0.44–11.82)	0.317	9.30 (0.90–96.19)	0.061

*H. pylori*								
Negative	Ref	—	Ref	—	Ref	—	Ref	—
Positive	**9.69 (2.31–40.50)**	**0.002**	**20.98 (4.42–99.47)**	**0.001**	**5.33 (1.10–25.81)**	**0.038**	—	0.988

Age								
≤45	Ref	—	Ref	—	Ref	—	Ref	—
45∼54	0.30 (0.05–1.80)	0.187	0.30 (0.05–1.92)	0.205	**0.10 (0.01–0.87)**	**0.037**	0.12 (0.01–2.53)	0.171
55∼64	0.73 (0.06–9.36)	0.808	0.37 (0.03–5.01)	0.453	1.13 (0.08–15.91)	0.922	1.40 (0.06–32.43)	0.833
≥65	0.16 (0.02–1.26)	0.083	**0.09 (0.01–0.76)**	**0.027**	0.42 (0.05–3.50)	0.420	—	0.989

miR-27a (*rs895819*) genotype								
TT	Ref	—	Ref	—	Ref	—	Ref	—
CT	1.02 (0.24–4.28)	0.976	1.10 (0.25–4.80)	0.898	1.72 (0.36–8.19)	0.495	4.41 (0.45–42.79)	0.201
CC	—	—	—	—	—	—	—	—
T	Ref	—	Ref	—	Ref	—	Ref	—
C	1.13 (0.36–3.55)	0.840	1.02 (0.32–3.28)	0.971	0.79 (0.23–2.73)	0.714	0.44 (0.09–2.24)	0.326

All the bold values represent that the outcome was statistically significant.

## Data Availability

The data used to support the findings of this study are available from the corresponding author upon request.
